# Structure and functions of a multireplicon genome of Antarctic *Psychrobacter* sp. ANT_H3: characterization of the genetic modules suitable for the construction of the plasmid-vectors for cold-active bacteria

**DOI:** 10.1007/s13353-023-00759-7

**Published:** 2023-05-05

**Authors:** Przemyslaw Decewicz, Krzysztof Romaniuk, Adrian Gorecki, Monika Radlinska, Maria Dabrowska, Agnieszka Wyszynska, Lukasz Dziewit

**Affiliations:** 1 Department of Environmental Microbiology and Biotechnology, Institute of Microbiology, Faculty of Biology, University of Warsaw, Warsaw, Poland; 2 Flinders Accelerator for Microbiome Exploration, College of Science and Engineering, Flinders University, Adelaide, Australia; 3 Department of Biochemistry and Microbiology, Institute of Biology, Warsaw University of Life Sciences (SGGW), Warsaw, Poland; 4 Department of Bacterial Genetics, Institute of Microbiology, Faculty of Biology, University of Warsaw, Warsaw, Poland

**Keywords:** Antarctica, Multireplicon genome, Plasmid, *Psychrobacter*, Vector

## Abstract

Among *Psychrobacter* spp., there are several multireplicon strains, carrying more than two plasmids. *Psychrobacter* sp. ANT_H3 carries as many as 11 extrachromosomal replicons, which is the highest number in *Psychrobacter* spp. Plasmids of this strain were subjected to detailed genomic analysis, which enables an insight into the structure and functioning of this multireplicon genome. The replication and conjugal transfer modules of ANT_H3 plasmids were analyzed functionally to discover their potential for being used as building blocks for the construction of novel plasmid-vectors for cold-active bacteria. It was shown that two plasmids have a narrow host range as they were not able to replicate in species other than *Psychrobacter*, while remaining plasmids had a wider host range and were functional in various *Alpha*- and *Gammaproteobacteria*. Moreover, it was confirmed that mobilization modules of seven plasmids were functional, i.e., could be mobilized for conjugal transfer by the RK2 conjugation system. Auxiliary genes were also distinguished in ANT_H3 plasmids, including these encoding putative DNA-protecting protein DprA, multidrug efflux SMR transporter of EmrE family, glycine cleavage system T protein, MscS small-conductance mechanosensitive channel protein, and two type II restriction-modification systems. Finally, all genome-retrieved plasmids of *Psychrobacter* spp. were subjected to complex genome- and proteome-based comparative analyses showing that Antarctic replicons are significantly different from plasmids from other locations.

**Supplementary Information:**

The online version contains supplementary material available at 10.1007/s13353-023-00759-7.

## Introduction

Antarctica is known to be one of the coldest regions on Earth, characterized also with increased UV radiation, strong winds, and the low amount of easily accessible nutrients (Martianov and Rakusa-Suszczewski 1990; Cowan et al. 2014). Antarctic bacteria are well-adapted, poly-extremophiles that can survive a variety of harsh physical and chemical conditions (Cowan et al. 2014). In our previous study, we obtained a collection of bacterial strains isolated from soil samples gathered on King George Island. This region is typical for Antarctica and is characterized by extreme conditions, including permanent cold with the average temperature of 2.0 °C, high humidity (78.7% in average), and average wind speed of 4.5 m∙s^−1^ (Araźny et al. 2013). Analyzed strains differed in their physiological features (including temperature and metals tolerance) and taxonomy, with three dominant genera: *Psychrobacter* (69 isolates), *Pseudomonas* (57), and *Arthrobacter* (47) (Romaniuk et al. 2018).

*Psychrobacter* spp. are Gram-negative, rod-shaped, non-motile, aerobic, heterotrophic bacteria frequently isolated from various habitats in Arctic and Antarctica. They are usually psychrotolerant, halotolerant, and non-pathogenic (Ayala-del-Rio et al. 2010). This group of bacteria is thoroughly studied, since they are recognized as model psychrotolerant microorganisms. *Psychrobacter* spp. are sources of cold-active enzymes for biotechnology, e.g., lipase (Esakkiraj et al. 2022), RNase (Wang et al. 2019), and esterase (Wu et al. 2015). Currently, over 250 complete and draft genomes of *Psychrobacter* spp. were obtained; however, up to date, much less is known about their plasmids. These are mostly small, cryptic replicons; however, in several of them, phenotypic modules were identified and their functionality was experimentally confirmed, including (i) fimbriae synthesis via the chaperone-usher pathway and genes involved in the aerobic and anaerobic metabolism of carnitine—pP32BP2 of *Psychrobacter* sp. DAB_AL32B (Ciok et al. 2019), (ii) laccase gene—pPspP11F6a of *Psychrobacter* sp. P11F6 (Moghadam et al. 2016), (iii) β-lactam, streptomycin, and tetracycline resistance genes—pKLH80 (Petrova et al. 2014), and (iv) organic sulfate metabolism operon—pP62BP1 of *Psychrobacter* sp. DAB_AL62B (Lasek et al. 2012). Only few *Psychrobacter* plasmids were used for the construction of novel vectors for molecular biology and biotechnology, while they are highly needed since *Psychrobacter* spp. are reference cold-active bacteria and potentially good candidates for developing effective expression systems active at low temperatures (Lasek et al. 2017; Ciok and Dziewit 2019).

Among *Psychrobacter* spp., there are several multireplicon strains, including *Psychrobacter* sp. P11G5 (seven plasmids; 5,511–40,963 bp) (Moghadam et al. 2016), *Psychrobacter* sp. 4Bb (six plasmids; 2,151–20,934 bp) (GenBank accession number: NZ_PJAS01000000), and *Psychrobacter* sp. Sarcosine-02u-2 (six plasmids; 1,929–11,462 bp) (GenBank accession number: NZ_PJBX00000000). However, the functioning of these multireplicon genomes was not investigated previously. In this study, we structurally and functionally characterized the plasmidome of a “record holder” *Psychrobacter* sp. ANT_H3, carrying 11 plasmids (3,124–13,249 bp). Moreover, we analyzed in detail replication and conjugal transfer modules of ANT_H3 plasmids to discover their potential for being used as building blocks for the construction of novel plasmid-vectors.

## Materials and methods

### Bacterial strains, plasmids, and culture conditions

The following laboratory bacterial strains were used: *Achromobacter* sp. LM16R (Dziewit et al. 2015), *Agrobacterium tumefaciens* LBA288 (Hooykaas et al. 1980), *Escherichia coli* BR825 (Ludtke et al. 1989), *E. coli* DH5α (Hanahan 1983), *E. coli* DH5αR (Bartosik et al. 2002), *E. coli* ER2566 (Zhou et al. 2020), *E. coli* S17-1 (Simon et al. 1983), *Paracoccus alcaliphilus* JCM 7364R (Bartosik et al. 2002), *Pseudomonas aeruginosa* PAO1161 (Bartosik et al. 2014), *Pseudomonas* sp. LM7R (Dziewit et al. 2013b), and *Variovorax paradoxus* EPS (Jamieson et al. 2009). The strains were grown on LB medium at 30 °C (EPS, JCM 7364R, LBA288, LM7R and LM16R) or 37 °C (BR825, DH5α, DH5αR, ER2566, and PAO1161). Where necessary, the medium was supplemented with X-gal, IPTG, and antibiotics: kanamycin (50 μg/ml for BR825, DH5α, EPS, JCM 7364R, LBA288, LM7R, or 500 μg/ml for LM16R and PAO1161), rifampicin (50 μg/ml), and streptomycin (50 μg/ml). The following vectors were used in this study: pABW1 (Bartosik et al. 1997)—for the functional analysis of the replication (REP) modules and pBGS18 (Spratt et al. 1986)—for the functional analysis of the mobilization (MOB) for conjugal transfer modules. Plasmids constructed in this study are listed in Supplementary Table S1.

### DNA manipulations and introduction of plasmid DNA into bacterial cells

The DNA of the plasmids was isolated using a GeneMATRIX Plasmid Miniprep DNA Purification Kit (EURx, Gdansk, Poland) or the classical alkaline lysis procedure. Routine DNA manipulations (including cloning of fragments of DNA, DNA digestion, and electrophoresis) were carried out using standard molecular biology methods (Sambrook and Russell 2001). DNA was amplified by PCR using a KAPA HiFi PCR Kit (KAPABIOSYSTEMS, Cape Town, South Africa) and appropriate primer pairs (Supplementary Table S2). DNA amplification was performed using a Mastercycler (Eppendorf, Hamburg, Germany). Each amplification started with an initial denaturation at 95 °C for 3 min followed by 30 cycles of denaturation at 98 °C for 20 s, annealing at 65 to 79 °C (depending on the primer pair) for 15 s, extension at 72 °C for 1 min/kb, and finished with a final extension at 72 °C for 1 min/kb. The PCR-amplified DNA fragments were then cloned in pABW1 or pBGS18 vectors.

Plasmids constructed in this study were introduced into *Achromobacter* sp. LM16R, *A. tumefaciens* LBA288, *P. alcaliphilus* JCM 7364, *Pseudomonas* sp. LM7R and *V. paradoxus* EPS by triparental mating, and *E. coli* DH5αR by biparental mating (Sambrook and Russell 2001). Additionally, constructed plasmids were introduced into *E. coli* BR825, DH5α, ER2566, and S17-1 and *P. aeruginosa* PAO1161R by chemical transformation (Kushner 1978; Irani and Rowe 1997).

### Testing of the host range of ANT_H3 plasmids

Derivatives of pABW1 carrying replication modules (REP) of ANT_H3 plasmids (Supplementary Table 1) were introduced into representatives of *Alphaproteobacteria* (*A. tumefaciens* LBA288 and *P. alcaliphilus* JCM 7364), *Betaproteobacteria* (*V. paradoxus* EPS i *Achromobacter* sp. LM16), and *Gammaproteobacteria* (*E. coli* BR825, *Pseudomonas* sp. LM7R, and *P. aeruginosa* PAO1161R). The ColE1-type replication system of pABW1 is not functional in any of these recipient strains (*E. coli* BR825 carries a mutation within the DNA polymerase I gene preventing ColE1-type replication). Thus, maintenance of the shuttle plasmids (pABW1-derivatives with various REP modules) in the tested hosts was fully dependent on the functions encoded within the cloned, predicted replication modules of the analyzed ANT_H3 plasmids. The presence of an introduced shuttle vector within a tested host strain was confirmed by the isolation of plasmid DNA from the recipient strain and subsequent electrophoresis of the isolation products.

### Testing the activity of the conjugal transfer systems

Derivatives of pBGS18 carrying conjugal transfer mobilization modules (MOB) of ANT_H3 plasmids (Supplementary Table 1) were introduced into *E. coli* S17-1 carrying chromosomally integrated *tra* genes. Biparental mating experiments using *E. coli* DH5αR as a recipient strain were performed for testing the activity of conjugal transfer systems of analyzed plasmids. The presence of an autonomous form of the introduced plasmid was confirmed by the isolation of plasmid DNA, its electrophoresis in 0.8% agarose gel, and the restriction analysis of isolated plasmid DNA.

### Testing of putative DNA methyltransferase activities

The predicted DNA methyltransferase genes identified within plasmids pA3H9 and pA3H10 were amplified using specific primer pairs (Supplementary Table S2). Then, the purified PCR products were digested with appropriate restriction enzymes and ligated with pET30a vector cut with the same enzymes. Resulting recombinant plasmids are listed in Supplementary Table S1. The recombinant methylases were expressed in the *E. coli* ER2566. Protein expression and restriction enzyme digestion protection assay revealing the sequence specificity of the methylase was performed as previously described (Drozdz et al. 2012).

### BIOLOG phenotyping assays

*Psychobacter* sp. ANT_H3 was tested for their utilization of various carbon and nitrogen sources using BIOLOG Phenotype MicroArrays (plates PM1, PM2A and PM3B) (Biolog, Inc.; Hayward, CA, USA) performed in duplicate. Plates were inoculated with bacteria that had been cultured on LA for 48 h and suspended in GN/GP fluid with Dye Mix G. The results were read after incubation at 20 °C for 96 h.

### DNA sequencing

DNA sequencing was performed using an Illumina MiSeq instrument in paired-end mode using a v3 chemistry kit. The obtained sequence reads were filtered for quality and assembled using Newbler v3.0 software (Roche, Basel, Switzerland). Final gap closure and sequencing of the cloned DNA fragments (for checking the constructed plasmids) was performed by the capillary sequencing of PCR products using an ABI3730xl DNA Analyzer (Applied Biosystems, Waltham, USA) applying primer walking technique.

### Bioinformatics

Plasmids were manually annotated using the Artemis software (Carver et al. 2012). Similarity searches were performed using the BLAST programs (Altschul et al. 1997) and Pfam tool (Finn et al. 2016) provided by the NCBI (http://blast.ncbi.nlm.nih.gov/Blast.cgi). The detection of tRNA sequences was performed using tRNAscan-SE program (Lowe and Chan 2016). Helix-turn-helix motifs were identified using the helix-turn-helix DNA-binding motif prediction tool (Dodd and Egan 1990). EC numbers were assigned using the KEGG database (Kanehisa et al. 2017) and UniProt Knowledgebase (UniProtKB) (Pundir et al. 2017). The analyzed draft genome was automatically annotated using RAST (Aziz et al. 2008; Brettin et al. 2015) on the BV-BRC 3.6.8 (Wattam et al. 2017) web service and afterwards manually curated. The final annotation of the genome was performed by the NCBI Prokaryotic Genome Annotation Pipeline (PGAP) (Tatusova et al. 2016), including the completeness (99.39%) and contamination (1.88%) verification with CheckM analysis v1.2.0 (Parks et al. 2015). The occurrence of the restriction sites with genetic modules was checked using NEBcutter v3 (Vincze et al. 2003). The ANT_H3 plasmids were delineated and compared with each other using clinker tool v0.0.20 (Gilchrist and Chooi 2021). All of the above analyses were performed using default parameters of each tool. Mobilization proteins, toxin-antitioxon system, and metal metabolism and resistance genes were classified with MOBscan (Garcillán-Barcia et al. 2020), TADB2 (Xie et al. 2018), and METGeneDb (Dziurzynski et al. 2022) databases, respectively. Searches against the TADB2 and METGeneDb databases were performed with a standalone BLASTP v2.12.0 with 1e-2 e-value threshold (Camacho et al. 2009).

Genomic comparison was performed using plasmids recovered from *Psychrobacter* genomes downloaded from GenBank database on September 12, 2022 using NCBI datasets command line toolkit (the command: *datasets download genome taxon Psychrobacter –annotated –assembly-source genbank –include-gbff –filename ncbi_datasets_GenBank_Psychrobacter.zip*) and genomes for strains DAB_AL43B, WY6, and KCTC 72,983 with indicated plasmids were downloaded manually (Supplementary Table S3). Plasmids were compared on nucleotide and protein levels. The nucleotide-based comparison was performed with all against all nucleotide BLAST v2.12.0 + searches with 1e-50 e-value threshold and then visualized with Circos v0.69–8 (Krzywinski et al. 2009). Protein-based comparison included all against all using protein BLAST with the following thresholds: *e*-value of 1e-10 and at least 30% and 90% sequence identity and HSP coverage, respectively. These were then used for the construction of a protein similarity network where a single node represented a protein and edge corresponded to a common reciprocated similarity of two proteins above thresholds. The thickness of the edge reflected the product of sequence alignment identity and coverage. The network was created using self-written Python scripts (available at https://github.com/DEMBresearch/ANT_H3_plasmids_genomics) and visualized in Gephi v0.9.7 using the Fruchterman-Reingold force-directed layout (Fruchterman and Reingold 1991; Bastian et al. 2009).

## Results

### Characterization of *Psychrobacter* sp. ANT_H3 and identification of its plasmids

*Psychrobacter* sp. ANT_H3 originated from petroleum-contaminated soil collected near the petroleum pumping and storage warehouse at the Henryk Arctowski Polish Antarctic Station (GPS coordinates: 62° 09.601′ S, 58° 28.464′ W). This is a psychrotolerant strain, growing in temperatures ranging from 4 to 37 °C, with an optimal temperature of growth at 22 °C. It tolerates a broad range of pH (from 5 to 11) and salinity up to 8%. The ANT_H3 strain exhibits a medium level (MIC = 4 mM) resistance to Cu(II) and low level (MIC = 2 mM) resistance to Ni(II) and Zn(II) (Romaniuk et al. 2018).

Testing of *Psychobacter* sp. ANT_H3 for its ability to utilize various carbon and nitrogen sources performed using BIOLOG Phenotype MicroArrays revealed that of the 190 carbon sources tested, the strain was able to use 27, including: acetic, glutamic, pyruvic, butyric, β-hydroxybutyric and α-3-methyl-2-oxobutanoic acids, Tween 20, Tween 40, Tween 80, L-glutamine, and L-histidine. In the case of nitrogen sources, activity was demonstrated for 15 compounds out of 95 tested, and the strain coped best with the decomposition of: glutamic acid, L-cysteine, L-glutamine, L-tryptophan, and the dipeptides: Ala-Gln and Ala-Glu.

**Fig. 1:**
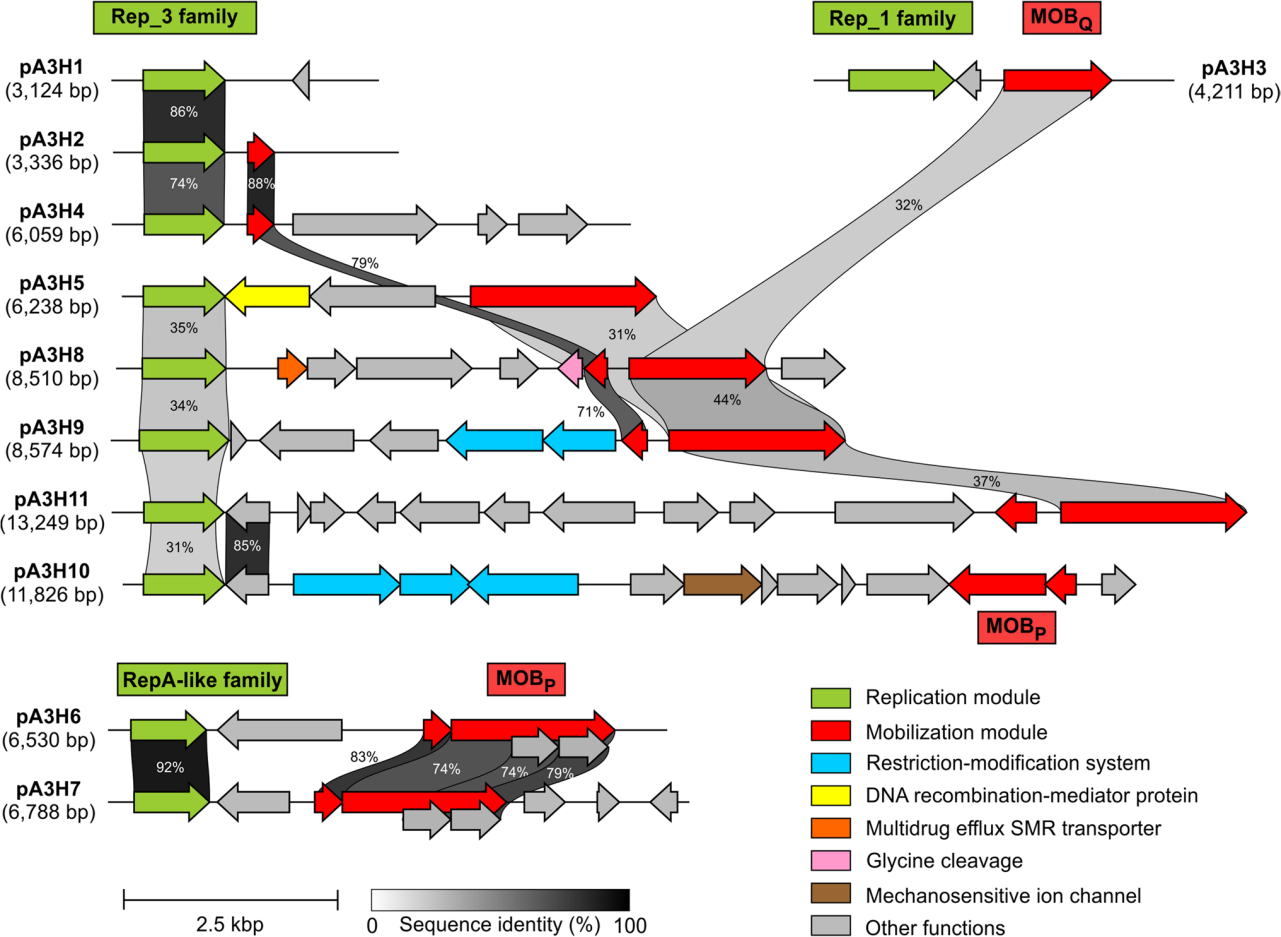
Linear presentation of the ANT_H3 plasmids. Each horizontal line represents plasmid and each arrow corresponds to the protein-encoding gene. If two arrows are connected with a gray-scale block, they show at least 30% sequence similarity as provided by clinker tool. Proteins belonging to the certain functional module were colored as indicated in the legend

The plasmid screening (applying alkaline lysis) revealed the presence of multiple plasmids. All plasmids isolated via alkaline lysis were subjected to DNA sequencing. The plasmids assembly revealed the presence of 11 plasmid contigs. After the final gap closure and sequencing of the cloned DNA fragments, it was revealed that plasmids ranged in their sizes between 3,124 and 13,249 bp, and they carried between 2 and 14 genes (Table 1 and Fig. 1).

**Table 1:** General features of the *Psychrobacter* sp. ANT_H3 plasmids

Plasmid	GenBank accession number	Plasmid size (bp)	GC content (%)	No. of genes	Predicted genetic modules^1^
pA3H1	MN657079	3,124	36.7	2	REP
pA3H2	MN657082	3,336	35.4	2	REP, MOB
pA3H3	MN657083	4,211	40.0	3	REP, MOB
pA3H4	MN657084	6,059	35.2	5	REP, MOB
pA3H5	MN657085	6,238	38.2	4	REP, MOB, DPR
pA3H6	MN657086	6,530	41.7	6	REP, MOB
pA3H7	MN657087	6,788	41.3	7	REP, MOB
pA3H8	MN657088	8,510	41.3	9	REP, MOB, SMR, GCV
pA3H9	MN657089	8,574	36.4	8	REP, MOB, RM
pA3H10	MN657080	11,826	43.4	14	REP, MOB, RM, MSC
pA3H11	MN657081	13,249	40.9	13	REP, MOB, MRS

^1^*DPR*, DNA recombination-mediator protein A; *GCV*, glycine cleavage; *MOB*, mobilization to conjugal transfer; *MRS*, multimer resolution system; *MSC*, mechanosensitive ion channel; *REP*, replication; *RM*, restriction–modification; *SMR*, multidrug efflux SMR transporter

Parallel to targeted isolation and sequencing of the ANT_H3 plasmids, the whole genome of this strain was subjected to sequencing using the Illumina MiSeq platform. This generated 1,485,328 paired reads and 446 Mbp of sequence information. As a result of the assembly of ANT_H3 genome, 65 contigs of a total length of 3,256,779 bp were obtained. The genome sequence was automatically annotated using RAST (Brettin et al. 2015) on the BV-BRC 3.28.9 web service (Olson et al. 2023), and its general features are presented in Table 2. With this genome sequencing, it was ultimately confirmed that the strain carries only 11 abovementioned plasmids and any mega-sized plasmid that could be skipped during alkaline lysis is present.

**Table 2:** General features of the *Psychrobacter* sp. ANT_H3 draft genome

Feature	Calculation
Strain	ANT_H3
Number of contigs	65
Estimated genome size (bp)	3,256,779
GC content (%)	42.96
Number of genes	2,905
Number of proteins with functional assignments	2,064
Number of proteins with EC number assignments	774
Number of tRNA genes	42
Number of regulatory RNA genes	3

### Replication modules (REP)

Bioinformatic analysis of the ANT_H3 plasmids revealed the presence of three types of REP modules: (i) Rep_3 family (*repB*-like)—pA3H1, pA3H2, pA3H4, pA3H5, pA3H8, pA3H9, pA3H10, and pA3H11; (ii) *repA*-like replicase—pA3H6 and pA3H7; (iii) Rep_1 rolling circle replication protein (Rep63 protein)—pA3H3.

The structure of the REP module of plasmids pA3H1, pA3H2, pA3H4, pA3H5, pA3H8, pA3H9, pA3H10, and pA3H11 is typical for the replication systems of many theta-replicating plasmids (Chattoraj 2000; Konieczny et al. 2014), i.e., they carry a gene encoding initiator of plasmid replication (Rep_3 family; RepB-like; pfam01051; COG5527), exhibiting nicking-closing (topoisomerase I-like) activity. The closely related replication protein is encoded by plasmid 1 (GenBank accession number: NC_007968) of the type-strain *Psychrobacter cryohalolentis* K5. Additionally, each REP module of abovementioned ANT_H3 plasmids contains also a predicted origin of replication (*oriV*), localized upstream of the *rep* gene, that is composed of four tandemly placed: (i) 22-bp-long iterons [5′-CATAAAGCTACGTTTAGCGACC-3′ for pA3H1; 5′-CATACCCTTACGTTTAGCGACC-3′ for pA3H2; 5′-CATACCACTACGTCTATCGACC-3′ for pA3H4; 5′-CATATGACGACAAATTCCTACC-3′ for pA3H8; 5′-CT(A/T)(T/G)ATGACTACAAATCCTTAC-3′ for pA3H9; 5′-(G/C)ATA(A/G)(A/G)(C/T)ATACGTTTATACACC-3′ for pA3H10] or (ii) 21-bp-long imperfect iterons [5′-(A/T)(A/T)(A/G)AAGCTAC(A/G)(A/T)(C/T)(A/T)ATCATA(A/G)-3′ for pA3H5] or 20-bp-long iterons [5′-AGAAAAAACAGTAAATGAGC-3′ for pA3H11] that may constitute binding sites for the Rep protein.

The second group of REP modules constitutes these found in pA3H6 and pA3H7 replicons. They contain a single gene encoding a replication protein (RepA-like) with three conserved regions, i.e., (i) the replicase domain (pfam03090), (ii) an alpha helical domain, found in the C-terminal parts of PriCT-1 primases, and (iii) a helix-turn-helix (HTH) motif responsible for interaction of Rep protein with DNA. These replication proteins exhibit amino acid sequence homology with the RepA protein of *E. coli* plasmid ColE2 (Yasueda et al. 1989). Moreover, in REP modules of plasmids pA3H6 and pA3H7, three DNA regions typical for the origin of replication of the ColE2 plasmid were found. These are (i) two direct repeats (L and R) with a reference consensus sequence 5′-CAGATAA-3′, (ii) sites α and β determining the specificity of the interactions of Rep protein with *oriV*, and (iii) a short sequence (5′-AGA-3′), where Rep protein synthesizes an unique RNA primer for the subsequent leading-strand DNA synthesis (Yagura et al. 2006).

The last type of the replication system was found in the pA3H3 plasmid, which encodes a Rep_1-type rolling circle replication protein (Rep63 protein). This mechanism of replication relies on generating one double-stranded plasmid and one circular ssDNA “plasmid” (that is later-on used as a matrix for the synthesis of the complementary strand) by a combined interaction of the plasmid-encoded Rep protein and the host DNA replicase (Ruiz-Masó et al. 2015). The Rep protein of pA3H3 plasmid exhibits highest similarity to the replication protein of *Psychrobacter alimentarius* (GenBank accession number: WP_201510399).

Following sequence analyses, REP modules of each ANT_H3 plasmid were PCR amplified and cloned into the pABW1 vector. This enabled testing their ability to replicate in bacteria belonging to different classes of Proteobacteria. None of the replication systems was functional in *Betaproteobacteria*. The replication systems of the pA3H3 and pA3H11 plasmids appeared to have a narrow host range as they were not able to replicate in species other than *Psychrobacter*. The remaining plasmids had a wider host range and, in addition to *Psychrobacter*, were functional in: (i) pA3H1—*P. aeruginosa* PAO1161; (ii) pA3H6—*A. tumefaciens* LBA288 and *P. alcaliphilus* JCM 7364R; (iii) pA3H2, pA3H5, pA3H7, and pA3H10—*E. coli*; and (iv) pA3H4, pA3H8, and pA3H9—*E. coli* and *Pseudomonas* spp.

### ANT-H3-originating REP modules as the building blocks for the construction of novel plasmid-vectors

After revealing the host range of the REP modules, their suitability for being used as the building blocks for the construction of novel plasmid-vectors functional in cold active bacteria was tested. This approach seems to be especially desired in modern biotechnology since psychrotolerant bacteria are becoming the novel type of microbial cell factories to produce cold-active enzymes and secondary metabolites (Santiago et al. 2016; Sonkar and Singh 2020; Styczynski et al. 2022b). What is more, it has been shown that there is a need to develop multi-plasmid systems, which makes cloning of several genes simultaneously (in one host) possible (Lasek et al. 2017; Ciok and Dziewit 2019).

For that purpose, each REP module (i.e., DNA region cloned in pABW1) was *in silico* checked for the presence of restriction sites most commonly occurring in multiple cloning sites (MCS) used for the construction of plasmid-vectors, i.e., sites recognized by the following 13 endonucleases: BamHI, EcoRI, EcoRV, HindIII, KpnI, NcoI, NheI, PstI, SacI, SalI, SphI, XbaI, and XhoI. Usually, only the modules that are poor in restriction sites are suitable for creation of novel vectors, since it gives the user a better possibility to use more MCS-localized restriction sites. As revealed, in the case of all REP modules of the ANT_H3 plasmids, majority, i.e., between 9 and 12 out of 13 tested restriction sites were available and thus these modules could be successfully used for the construction of the novel plasmid-vectors in the future (Table 3).

**Table 3:** Occurrence of BamHI-, EcoRI-, EcoRV-, HindIII-, KpnI-, NcoI-, NheI-, PstI-, SacI-, SalI-, SphI-, XbaI-, and XhoI-specific restriction sites within REP modules of ANT_H3 plasmids

Plasmid harboring the REP module	Tested DNA region (coordinates/GenBank accession number)	Restriction enzymes NOT cutting within the tested genetic module
REP of pA3H1	2,670–2,399/MN657079	BamHI, EcoRI, KpnI, NcoI, NheI, PstI, SacI, SalI, SphI, XbaI, XhoI
REP of pA3H2	2,964–2,348/MN657082	BamHI, EcoRI, EcoRV, KpnI, NcoI, NheI, PstI, SacI, SalI, SphI, XbaI, XhoI
REP of pA3H3	3,957–1,854/MN657083	BamHI, EcoRI, KpnI, NcoI, NheI, SacI, SalI, SphI, XbaI, XhoI
REP of pA3H4	5,628–1,728/MN657084	BamHI, EcoRI, EcoRV, KpnI, NcoI, SacI, SalI, XbaI, XhoI
REP of pA3H5	6,087–1,326/MN657085	BamHI, EcoRI, EcoRV, HindIII, KpnI, NcoI, NheI, SacI, SalI, SphI, XbaI, XhoI
REP of pA3H6	6,396–1,430/MN657086	BamHI, EcoRI, EcoRV, KpnI, NcoI, NheI, PstI, SacI, SalI, XbaI, XhoI
REP of pA3H7	6,363–1,532/MN657087	BamHI, EcoRI, EcoRV, KpnI, NcoI, PstI, SacI, SalI, SphI, XbaI, XhoI
REP of pA3H8	8,303–2,062/MN657088	BamHI, EcoRI, KpnI, NcoI, PstI, SacI, SalI, SphI, XhoI
REP of pA3H9	8,354–1,571/MN657089	BamHI, EcoRI, HindIII, KpnI, NcoI, NheI, PstI, SacI, SalI, XbaI, XhoI
REP of pA3H10	11,689–1,350/MN657080	BamHI, EcoRV, KpnI, NcoI, NheI, SacI, SalI, XbaI, XhoI
REP of pA3H11	13,052–1,491/MN657081	BamHI, EcoRI, EcoRV, KpnI, NcoI, NheI, SacI, SalI, SphI, XbaI, XhoI

### Mobilization to conjugal transfer (MOB) modules

In 10 of ANT_H3 plasmids, modules for mobilization to conjugal transfer were identified. Such a module was not present in the smallest identified replicon—pA3H1. Two plasmids (pA3H2 and pA3H4) contained orphan *mobC*-like gene and possibly *oriT*s, while remaining plasmids carried classical gene clusters encoding relaxases (MobA). Based on amino acid sequence similarities of the relaxases, they were classified into two distinct families, namely, MOB_P_ and MOB_Q_ (Garcillán-Barcia et al. 2020).

The MOB_P_ family members were found in pA3H6, pA3H7, and pA3H10. All three proteins exhibited the highest similarity to various relaxases of *Psychrobacter* spp. It was also revealed that enzymes of pA3H6 and pA3H7 showed high reciprocal similarity level (74%), while the relaxase of pA3H10 was more diverse, showing only about 34% of similarity (on the part of the sequence) to the other two.

In five plasmids, namely, pA3H3, pA3H5, pA3H8, pA3H9, and pA3H11, relaxases of the MOB_Q_ family were encoded. This was the most abundant and diverse group of mobilization systems (Francia et al. 2004; Garcillan-Barcia et al. 2009). Analyzed relaxases showed highest similarity to appropriate proteins of *Psychrobacter* spp. and *Moraxella* spp. Interestingly, although all these relaxases belonged to the same family, only two of them (i.e., encoded within pA3H8 and pA3H9) showed high level of reciprocal similarity (87%), while others were more diverse, with similarities less than 50%.

The functionality of the MOB modules was examined. Each module was cloned into pBGS18, and then conjugation tests were performed using plasmid pRK2013 (RK2 conjugation transfer system) as the mobilization agent. It was confirmed that MOB modules of seven plasmids: pA3H4, pA3H5, pA3H6, pA3H7, pA3H8, pA3H9, and pA3H11 were functional, i.e., could be mobilized for conjugal transfer by the RK2 conjugation system. The remaining three systems revealed to be inactive, which suggested that they were non-functional or could not be mobilized to conjugal transfer by pRK2013.

### Auxiliary genetic information

Within the analyzed plasmids, besides modules comprising conserved plasmid backbone (i.e., REP and MOB), a putative function was predicted for only several other genes. However, their biological activity was mostly based on *in silico* analyses and would need further investigation for proving.

Among these genes were (i) *H5F75_RS00010* of pA3H5 that encoded a predicted DNA-protecting protein DprA (COG0758) that protects incoming foreign DNA and thus is active in many bacteria naturally competent for transformation of DNA, plus it is an accessory factor for RecA-mediated DNA strand exchange (Hovland et al. 2017); (ii) *H5F64_RS00010* of pA3H8 encoding predicted multidrug efflux SMR transporter of EmrE family (COG2076) that may be involved in export of a range of toxins, including ethidium bromide and quaternary ammonium compounds (Ovchinnikov et al. 2018); (iii) *H5F64_RS00020* of pA3H8 encoding the predicted glycine cleavage system T protein (aminomethyltransferase) (COG0404) that is a part of the glycine cleavage multienzyme complex (GCV) found in bacteria and the mitochondria of eukaryotes (Kikuchi et al. 2008); and (iv) *H5F49_RS00035* of pA3H10 encoding a putative MscS small-conductance mechanosensitive, anion- or cation-selective channel protein (COG0668) opening in response to stretch forces in the membrane lipid bilayer (Martinac 2001).

Additionally, in two plasmids (pA3H9 and pA3H10), the putative type II restriction-modification systems were identified. Their function may be linked with cell defense against exogenous DNA or stable maintenance of plasmids in the host cell (Vasu and Nagaraja 2013). The pA3H9 plasmid system was composed of two non-overlapping genes (*H5F60_RS00025* and *H5F60_RS00030*), encoding predicted restriction endonuclease and adenine methyltransferase. In the case of plasmid pA3H10, the potential restriction-modification system was more complex. It consisted of two genes encoding adenine methyltransferases (*H5F49_RS00015* and *H5F49_RS00020*) and a complementary-strand-encoded restriction endonuclease (*H5F49_RS00025*). The first of these two methyltransferases showed sequence similarity to 5′-GAATTC-3′-specific methylases, e.g., 40% sequence identity with M.EcoRI (GenBank accession number: AAA26372) encoded by the plasmid RI13 from *E. coli* C600. Functional analysis of the methyltransferases was performed using a restriction enzyme digestion protection assay (Drozdz et al. 2012). The experimental set-up allowed determining the specificity of the methylase of pA3H9, which recognized the 5′-GAWTC-3′ sequence. Additionally, as the DNA of pET-MET10a and pET-MET10b (carrying methyltransferases of pA3H10) were cleaved by all restriction enzymes used in the test known to be insensitive to adenine methylation in their cognate sequences (including EcoRI), we hypothesize that for the full methylation the presence of both enzymes in the cell is needed.

### Comparative analyses of the *Psychrobacter* plasmids

**Fig. 2:**
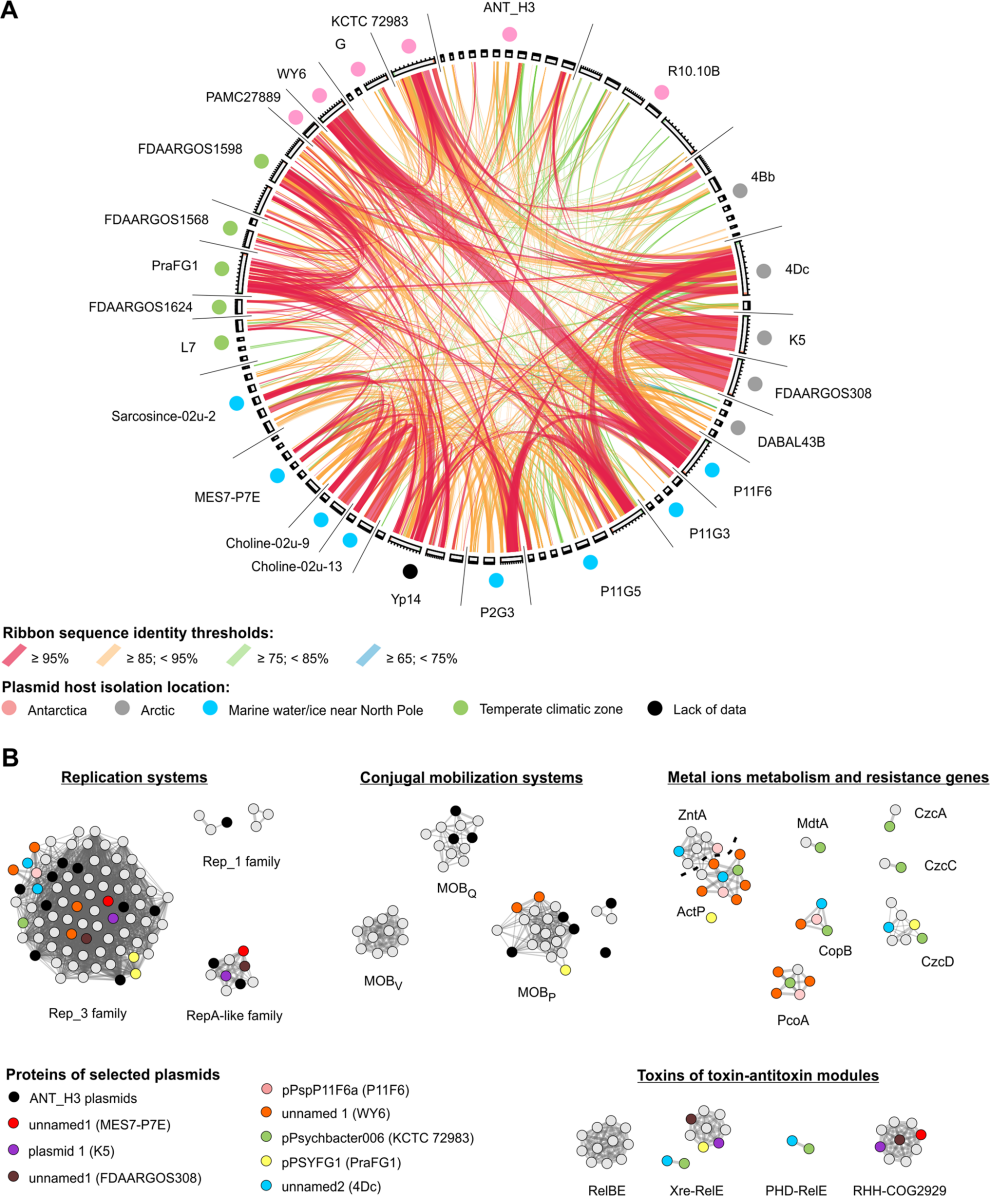
Comparative genomics of plasmids of *Psychrobacter* spp. with assembled genomes. Panel A presents nucleotide-based sequence similarity of *Psychrobacter* plasmids. Their clockwise order corresponds to the order in Supplementary Table S3. Panel B presents a protein-based similarity network where each node corresponds to a single protein and an edge connecting two nodes represents sequence similarity between two proteins above the given thresholds. Four functional groups of proteins were presented. Proteins encoded within the selected plasmids were color-coded leaving other proteins gray

In total, 74 plasmids were distinguished in 24 out of 124 publicly available *Psychrobacter* spp. genomes (Supplementary Table S3). They ranged in size from 1,839 to 59,859 bp. These plasmids (together with ANT_H3 plasmids) were used for the comparative genomic and proteomic analyses. Ten plasmids (carried by six strains), including pA3H3 and pA3H5, seem to be unique as they did not show any sequence similarities to other analyzed plasmids. Remaining ANT_H3 plasmids showed limited sequence homology (Fig. 2A) with other *Psychrobacter* plasmids. This can be recognized as a common feature of all *Psychrobacter* plasmids, since, in general, only short DNA segments of *Psychrobacter* plasmids were identical or highly similar between various replicons originating from different locations. Interestingly, the most diverse replicons were identified in Antarctic strains.

To further explore the common genetic load of these plasmids and to determine the groups of proteins shared among plasmids, a protein-based similarity network based on all against all protein BLAST search was constructed (Fig. 2B). This allowed determining that 68, 9, and 5 of *Psychrobacter* plasmids carry Rep3, RepA-like, and Rep_1 family replication system, respectively. For three plasmids, namely, unnamed6 (4Bb strain), unnamed1 (4Dc), and unnamed 5 (MES7-P7E), the replication system could not be determined. On the other hand, plasmids unnamed 1 (MES7-P7E), plasmid 1 (K5), and unnamed 1 (FDAARGOS_308) were the only composite replicons carrying two replication systems, here belonging to Rep3 and RepA-like families (Fig. 2B).

In the case of mobilization systems, within 44 plasmids from 18 strains, three groups of MOB families, namely, MOB_Q_ (23 representatives), MOB_P_ (12,) and MOB_V_ (11), were found (Fig. 2B). Plasmid 1 of R10.10B was the only plasmid encoding two nearly identical MOB_Q_ proteins (AOC03_11960 and AOC03_12020; 98% sequence identity), while plasmid 2 of that strain was also the only one with two different mobilization systems, i.e., MOB_P_ (AOC03_12040) and MOB_V_ (AOC03_12075). Moreover, from among other multireplicon strains, besides ANT_H3, only two carried plasmids with different mobilization systems, i.e., Sarcosine-02u-2 (unnamed 2—MOB_Q_, unnamed 3—MOB_V_, unnamed 4 and 5—MOB_P_) and DAB_AL43B (pP43BP1 and pP43BP4—MOB_P_, pP43BP2 and pP43BP3—MOB_V_).

Finally, when looking at the additional genetic load of *Psychrobacter* plasmids, there were two interesting groups of genes (Fig. 2B). The first one consists of 39 metal metabolism and resistance genes (MMRGs), involved in processing zinc and cadmium (ZntA), copper (ActP, CopB, and PcoA), zinc (MdtA), and cobalt, zinc, and cadmium (CzcA, CzcC, and CzcD). They were carried by 11 plasmids from 11 strains and except for the plasmid unnamed 2 (of MES7-P7Y) with the genome size of 6,585 bp, all these replicons were larger than 20 kb. Plasmids unnamed 1 (31,994 bp; WY6), pPsychbacter006 (49,070 bp; KCTC 72,983), unnamed 2 (59,859 bp; 4Dc), and pPspP11F6a (44,793 bp; P11F6) encode the highest number of MMRGs, i.e., 10, 7, 5, and 4, respectively.

Beside MMRGs, 28 plasmids from 17 *Psychrobacter* spp. encoded type II toxin-antitoxin systems, which could increase replicons’ stability in the host cells. They belonged to four families, i.e., RelBE (11 proteins), Xre-RelE (10), PHD-RelE (2), and RHH-COG2929 (9). The first three rely on RelE cytotoxin targeting ribosome and by that stopping the translational process (Christensen-Dalsgaard et al. 2010), while the RHH-COG2929 antitoxin-toxin pair mechanism remains undetermined.

## Discussion

Based on the physiological and metabolic characterization, Antarctic *Psychrobacter* sp. ANT_H3 turned out to be similar to other species of this genus, including Arctic strains (Dziewit et al. 2013a). The main difference, which was also encouraging for further genomic analyses, was the presence of as many as 11 plasmids in the ANT_H3 genome. This is a true record in *Psychrobacter* spp. Within the analyzed plasmids, several auxiliary genes of metabolic potential were found; however, it seems that they were inactive, e.g., as judged from the BIOLOG phenotyping assays the ANT-H3 strain could not utilize glycine thus the pA3H8-encoded predicted glycine cleavage system T protein (aminomethyltransferase) was rather non-functional.

It was shown that 14% (17 out of 125) of *Psychrobacter* strains with sequenced genomes carry at least two plasmids. Majority of these plasmids are small (< 10 kb), cryptic replicons. Therefore, it is highly intriguing what their biological functions are. If they are just the selfish DNAs or may contribute to the host’s evolution as the mobile platforms enabling expression of acquired genetic information or at least act as the vehicles of auxiliary genetic load in horizontal gene transfer. Investigation of the genetic content of these plasmids revealed the dominance (besides the REP and MOB modules) of genes encoding hypothetical proteins for which no function was predicted. This does not preclude the possibility that *Psychrobacter* plasmids carry genes impacting the host’s phenotype, but rather proofs that annotation of bacterial genomes still remains imperfect and manual data curation and improving the reference databases seems to be the only solution so far, although it is usually laborious and tedious (Petty 2010).

As mentioned above, *Psychrobacter* spp. represents a group of bacteria with multipartite genomes that carry mostly small, cryptic plasmids. In this group, also other bacteria can be included, e.g., same species of *Aeromonas* spp. (Attéré et al. 2017) and *Paracoccus* spp. (Maj et al. 2013). Plasmids of these bacteria are mostly of the narrow host range, but still contain MOB modules, which suggests the possibility of their mobilization for conjugal transfer. As shown previously, such plasmids are efficient carriers of genetic information, since they may act as natural suicide vectors “stimulating” incorporation of genes into hosts’ genomes even among evolutionarily distinct bacterial species (Smorawinska et al. 2012). In the case of the ANT_H3 strain, it was shown that some REP modules are functional in bacteria other than *Psychrobacter* spp., which suggests that they might be acquired in horizontal transfer of genes and may potentially invade novel hosts, crossing the taxonomic barrier.

From the biotechnological point of view, *Psychrobacter* spp. are important not only as native producers of various biologically active compounds (Styczynski et al. 2022a; D’Angelo et al. 2022) but also as potential heterological hosts for overexpression of genes of other bacteria, including psychrotolerant ones, like it was previously developed for *Pseudoalteromonas haloplanktis* TAC125 (Duilio et al. 2004). Using *Psychrobacter* spp. as cold-active expression systems may potentially improve purification of various proteins, since lowering temperature during overexpression is a common procedure during problematic purification procedures (Sambrook and Russell 2001). However, this is naturally limited by the temperature tolerance of *E. coli* or other bacteria used in the assays and may be overcome by using naturally psychrotolerant *Psychrobacter* spp. Planning genetic manipulations in this group of bacteria one has to be aware of the limited number of plasmid-vectors available. Thus, analyses of *Psychrobacter* plasmids are of high relevance, since this may deliver the building blocks, i.e., functional genetic modules, for the construction of novel vectors. In the case of the ANT_H3 strain, there is a unique situation in which we have as many as 11 plasmids co-occurring in one host, which enables using their compatible REP modules for the construction of multi-plasmid systems functional in *Psychrobacter* spp., and possibly in other cold-active bacteria.

In this study, a comparative genomic analysis was also performed which revealed the genome size and gene content variability of *Psychrobacter* plasmids. The most remote were plasmids from Antarctica. That exemplifies the uniqueness of Antarctic ecosystem, most probably caused by its geographic isolation (Doytchinov and Dimov 2022). Nevertheless, it was shown that the similar plasmids often shared short but nearly identical DNA segments, which included not only maintenance systems (replication, mobilization, or stabilization) but also auxiliary genes like MMRGs. It is somehow intriguing to observe that the majority of identified MMRGs were identified in one plasmid even though the given strain carried more plasmids. This suggests that certain replicons could specialize in accumulating this kind of genes. Interestingly, six of 11 MMRG-carrying plasmids were not self-transmissible (lack of MOB module). That raises questions about the evolution of such non-mobile replicons and the source of their auxiliary genes, as these could be acquired from other temporally carried plasmids or the host’s chromosome. It is also possible that non-mobile plasmids simply lost their mobility modules and became “domesticated,” giving their hosts environmental advantage.

## Supplementary Information

Below is the link to the electronic supplementary material.

Supplementary file1 (DOCX 22 KB)

## Data Availability

All data described in the manuscript are available either in Electronic Supplementary Materials associated with this manuscript or in genomic database. The draft genome sequence of *Psychrobacter* sp. ANT_H3 and complete nucleotide sequences of its plasmids were deposited in the GenBank (NCBI) database with the accession numbers: JAQISM000000000 and MN657079-MN657089, respectively.
