# Supplementary material for: Structure and functions of a multireplicon genome of Antarctic Psychrobacter sp. ANT_H3: characterization of the genetic modules suitable for the construction of the plasmid-vectors for cold-active bacteria
Source: J Appl Genet. 2023 May 5;64(3):545–57. doi: 10.1007/s13353-023-00759-7 (PMC10457243; doi:10.1007/s13353-023-00759-7)
Supplement: Supplementary file 1 — Supplementary file1 (DOCX 22 KB) [file 13353_2023_759_MOESM1_ESM.docx]

**Supplementary materials**

**Supplementary Table 1.** Plasmids constructed in this study.

| **Plasmid name** | **Characteristics*** |
| --- | --- |
| pABW1-REP1 | pABW1 carrying the REP module of pA3H1 (PCR-amplified with the primers LREP3H1 and RREP3H1) |
| pABW1-REP2 | pABW1 carrying the REP module of pA3H2 (PCR-amplified with the primers LREP3H2 and RREP3H2) |
| pABW1-REP3 | pABW1 carrying the REP module of pA3H3 (PCR-amplified with the primers LREP3H3 and RREP3H3) |
| pABW1-REP4 | pABW1 carrying the REP module of pA3H4 (PCR-amplified with the primers LREP3H4 and RREP3H4) |
| pABW1-REP5 | pABW1 carrying the REP module of pA3H5 (PCR-amplified with the primers LREP3H5 and RREP3H5) |
| pABW1-REP6 | pABW1 carrying the REP module of pA3H6 (PCR-amplified with the primers LREP3H6 and RREP3H6) |
| pABW1-REP7 | pABW1 carrying the REP module of pA3H7 (PCR-amplified with the primers LREP3H7 and RREP3H7) |
| pABW1-REP8 | pABW1 carrying the REP module of pA3H8 (PCR-amplified with the primers LREP3H8 and RREP3H8) |
| pABW1-REP9 | pABW1 carrying the REP module of pA3H9 (PCR-amplified with the primers LREP3H9 and RREP3H9) |
| pABW1-REP10 | pABW1 carrying the REP module of pA3H10 (PCR-amplified with the primers LREP3H10 and RREP3H10) |
| pABW1-REP11 | pABW1 carrying the REP module of pA3H11 (PCR-amplified with the primers LREP3H11 and RREP3H11) |
| pBGS-MOB2 | pBGS18 carrying the MOB module of pA3H2 (PCR-amplified with the primers LMOB3H2 and RMOB3H2) |
| pBGS-MOB3 | pBGS18 carrying the MOB module of pA3H3 (PCR-amplified with the primers LMOB3H3 and RMOB3H3) |
| pBGS-MOB4 | pBGS18 carrying the MOB module of pA3H4 (PCR-amplified with the primers LMOB3H4 and RMOB3H4) |
| pBGS-MOB5 | pBGS18 carrying the MOB module of pA3H5 (PCR-amplified with the primers LMOB3H5 and RMOB3H5) |
| pBGS-MOB6 | pBGS18 carrying the MOB module of pA3H6 (PCR-amplified with the primers LMOB3H6 and RMOB3H6) |
| pBGS-MOB7 | pBGS18 carrying the MOB module of pA3H7 (PCR-amplified with the primers LMOB3H7 and RMOB3H7) |
| pBGS-MOB8 | pBGS18 carrying the MOB module of pA3H8 (PCR-amplified with the primers LMOB3H8 and RMOB3H8) |
| pBGS-MOB9 | pBGS18 carrying the MOB module of pA3H9 (PCR-amplified with the primers LMOB3H9 and RMOB3H9) |
| pBGS-MOB10 | pBGS18 carrying the MOB module of pA3H10 (PCR-amplified with the primers LMOB3H10 and RMOB3H10) |
| pBGS-MOB11 | pBGS18 carrying the MOB module of pA3H11 (PCR-amplified with the primers LMOB3H11 and RMOB3H11) |
| pET-MET9 | pET30a carrying the DNA methyltransferase gene (PCR-amplified with the primers_F_NdeI and Met_A3H9R_XhoI) |
| pET-MET10a | pET30a carrying the DNA methyltransferase gene (PCR-amplified with the primers RelBMet_F_NdeI and RelBMet_R_XhoI) |
| pET-MET10b | pET30a carrying the DNA methyltransferase gene (PCR-amplified with the primers Met_A3H10_F_NdeI and Met_A3H10_R_SalI) |

* Primer sequences are listed in Supplementary Table S2; REP – replication, MOB – mobilization for conjugal transfer

**Supplementary Table 2.** PCR primers used in this study.

| **Primer name** | **Target plasmid** | **Sequence (5’-3’)*** |
| --- | --- | --- |
| LREP3H1 | pA3H1 | ttcgcgaattcCCATGCTCCTTCAGTTGCTTCC |
| RREP3H1 | pA3H1 | tccggatccACAAGAGAATGCCCTGGCTGTC |
| LREP3H2 | pA3H2 | tgcgcgaattcCCCAAGGGAACCAGTTGTTTAG |
| RREP3H2 | pA3H2 | ggccggatccCTAACAAGCACTCGGGATTACC |
| LREP3H3 | pA3H3 | tgcgcgaattcACGGCTATCTTAGCAACG |
| RREP3H3 | pA3H3 | ggccggatccATAACGGGCGGTATGATG |
| LREP3H4 | pA3H4 | tgcgcgaattcGTAACCAACCGTGAATCG |
| RREP3H4 | pA3H4 | ggccggatccCTCTAAAGCGTCCACAAG |
| LREP3H5 | pA3H5 | tgcgcgaattcAAAGCACTCAGAGGCTCTAC |
| RREP3H5 | pA3H5 | ggccggatccAAACGATTGAAGAGCATCAG |
| LREP3H6 | pA3H6 | tgcgcgaattcTGGAGGTTAGCAACTCCTAC |
| RREP3H6 | pA3H6 | ggccggatccACCAAGGCAATGCATCTG |
| LREP3H7 | pA3H7 | tgcgcgaattcGTCAAATTGGCACGACGTTC |
| RREP3H7 | pA3H7 | ggccggatccAAGGGCACTCGTAAATGCTCTG |
| LREP3H8 | pA3H8 | tgcgcgaattcAAGCTTGGGTGCAATGTG |
| RREP3H8 | pA3H8 | ggccggatccTAAGGTGCGTAATGCCTGTG |
| LREP3H9 | pA3H9 | tgcgcgaattcCTACGCAGTATCAGCTTG |
| RREP3H9 | pA3H9 | ggccggatccTCCCTGATTGGACGTTTC |
| LREP3H10 | pA3H10 | tgaacgagctcACTGCTGCTCAGCTAATG |
| RREP3H10 | pA3H10 | ggccggatccAAGCCAACGCTAGTATCC |
| LREP3H11 | pA3H11 | tgcgcgaattcTTGCGTGAGGTTAACTCC |
| RREP3H11 | pA3H11 | ggccggatccAAGCCAACGCTAGTATCC |
| LMOB3H2 | pA3H2 | tagcagaattcTGCCACTCCTGACTTGTTC |
| RMOB3H2 | pA3H2 | tccaggatccGGGCTAAACAACTGGTTC |
| LMOB3H3 | pA3H3 | tagcagaattcAGATGATGCGAGATGCTGAC |
| RMOB3H3 | pA3H3 | tccaggatccCGCTCAGACGGTAGTAATTTGC |
| LMOB3H4 | pA3H4 | tagcagaattcAATGACGGCTGACCAGTTAGGC |
| RMOB3H4 | pA3H4 | tccaggatccGCGCAAAGAGTTGGCTTGCATC |
| LMOB3H5 | pA3H5 | tggcagaattcCAACACCCGCTCTATAAG |
| RMOB3H5 | pA3H5 | tccaggatccCTTTGGCAACCCTAGTTC |
| LMOB3H6 | pA3H6 | tggcagaattcGTACTCTCGTTCAGCGACTTTC |
| RMOB3H6 | pA3H6 | tccactgcagAGCTCGCCACGTTTATCATC |
| LMOB3H7 | pA3H7 | tggcagaattcCACCTTGCTCAGCTTTACTC |
| RMOB3H7 | pA3H7 | tccaggatccTCGCCTTCGTAGTACATCAG |
| LMOB3H8 | pA3H8 | tggcagaattcTCTAATACCGCCATCTAGACCC |
| RMOB3H8 | pA3H8 | tccaggatccGAGAACTCATCACCAGCATCAG |
| LMOB3H9 | pA3H9 | tggcagaattcGCTATAGACAGCGCCATTCG |
| RMOB3H9 | pA3H9 | tccaggatccACTGCTCTGACGCTTCAC |
| LMOB3H10 | pA3H10 | tggcagaattcGCGCAACGAAATAACGACCATC |
| RMOB3H10 | pA3H10 | tccaggatccTTACACGCTGAGCGACTAAG |
| LMOB3H11 | pA3H11 | tggcagaattcATGATACAGGCCGCATAC |
| RMOB3H11 | pA3H11 | tccaggatccTTCGGGCAAGCTCATTAC |
| Met_A3H9_F_NdeI | pA3H9 | gttgttcatATGAGTACGAGAAAACCTGGATTATTG |
| Met_A3H9_R_XhoI | pA3H9 | gaatcactcgagATAATTCCTTATAACTAAATGATGTGCATCTTG |
| RelBMet_F_Nde | pA3H10 | gttgttcatATGTCCACTAAGCCAACTACGAC |
| RelBMet_R_XhoI | pA3H10 | gaatcactcgagAGATTTCCTGCGAATCATAATTCGC |
| Met_A3H10_F_NdeI | pA3H10 | gttgttcatATGAGTAGTTTAATGCGCGTTCC |
| Met_A3H10_R_SalI | pA3H10 | gaatcagtcGACGCGACTTAGCCACTCTTG |

* Sequences not complementary to the target plasmids are presented in lower case. Restriction sites used for cloning are underlined.

**Supplementary Table 3.** *Psychrobacter* plasmids included in comparative genomic analysis.

| **GenBank accession number** | **Plasmid name** | **Genome size (bp)** | **Number of genes** | **Host strain** |
| --- | --- | --- | --- | --- |
| CP014946.1 | pPAMC27889 | 16905 | 16 | *Psychrobacter alimentarius* |
| CP049903.1 | unnamed1 | 31994 | 53 | *Psychrobacter* sp. WY6 |
| CP006268.1 | PsyG_3 | 3956 | 5 | *Psychrobacter* sp. G |
| CP006267.1 | PsyG_4 | 4518 | 5 | *Psychrobacter* sp. G |
| CP006266.1 | PsyG_26 | 26087 | 26 | *Psychrobacter* sp. G |
| CP060391.1 | pPsychbacter006 | 49070 | 53 | Psychrobacter sp. KCTC 72983 |
| MN657079.1 | pA3H1 | 3124 | 2 | *Psychrobacter* sp. ANT_H3 |
| MN657082.1 | pA3H2 | 3336 | 2 | *Psychrobacter* sp. ANT_H3 |
| MN657083.1 | pA3H3 | 4211 | 3 | *Psychrobacter* sp. ANT_H3 |
| MN657084.1 | pA3H4 | 6059 | 5 | *Psychrobacter* sp. ANT_H3 |
| MN657085.1 | pA3H5 | 6238 | 4 | *Psychrobacter* sp. ANT_H3 |
| MN657086.1 | pA3H6 | 6530 | 6 | *Psychrobacter* sp. ANT_H3 |
| MN657087.1 | pA3H7 | 6788 | 9 | *Psychrobacter* sp. ANT_H3 |
| MN657088.1 | pA3H8 | 8510 | 9 | *Psychrobacter* sp. ANT_H3 |
| MN657089.1 | pA3H9 | 8574 | 8 | *Psychrobacter* sp. ANT_H3 |
| MN657080.1 | pA3H10 | 11826 | 14 | *Psychrobacter* sp. ANT_H3 |
| MN657081.1 | pA3H11 | 13249 | 13 | *Psychrobacter* sp. ANT_H3 |
| CP012707.1 | 1 | 24169 | 24 | *Psychrobacter urativorans* |
| CP012708.1 | 2 | 15605 | 12 | *Psychrobacter urativorans* |
| CP012709.1 | 3 | 24210 | 22 | *Psychrobacter urativorans* |
| CP012710.1 | 4 | 16222 | 12 | *Psychrobacter urativorans* |
| CP012711.1 | 5 | 49020 | 36 | *Psychrobacter urativorans* |
| CM009109.1 | unnamed1 | 20934 | 20 | *Psychrobacter* sp. 4Bb |
| CM009110.1 | unnamed2 | 15219 | 15 | *Psychrobacter* sp. 4Bb |
| CM009111.1 | unnamed3 | 5133 | 5 | *Psychrobacter* sp. 4Bb |
| CM009112.1 | unnamed4 | 4685 | 4 | *Psychrobacter* sp. 4Bb |
| CM009113.1 | unnamed5 | 4227 | 2 | *Psychrobacter* sp. 4Bb |
| CM009114.1 | unnamed6 | 2151 | 2 | *Psychrobacter* sp. 4Bb |
| CM009115.1 | unnamed1 | 1839 | 1 | *Psychrobacter* sp. 4Dc |
| CM009116.1 | unnamed2 | 59859 | 57 | *Psychrobacter* sp. 4Dc |
| CM009117.1 | unnamed3 | 8891 | 10 | *Psychrobacter* sp. 4Dc |
| CP000324.1 | 1 | 41221 | 44 | *Psychrobacter cryohalolentis* K5 |
| CP022042.2 | unnamed1 | 41221 | 47 | *Psychrobacter cryohalolentis* |
| JQ245700.1 | pP43BP1 | 4390 | 6 | *Psychrobacter* sp. DAB_AL43B |
| JQ245701.1 | pP43BP2 | 5445 | 6 | *Psychrobacter* sp. DAB_AL43B |
| JQ348845.1 | pP43BP3 | 4955 | 7 | *Psychrobacter* sp. DAB_AL43B |
| JQ348844.1 | pP43BP4 | 6450 | 9 | *Psychrobacter* sp. DAB_AL43B |
| CM003595.1 | pPspP11F6a | 44793 | 37 | *Psychrobacter* sp. P11F6 |
| CM003597.1 | pPspP11G3a | 8535 | 5 | *Psychrobacter* sp. P11G3 |
| CM003598.1 | pPspP11G3b | 6907 | 5 | *Psychrobacter* sp. P11G3 |
| CM003599.1 | pPspP11G3c | 5621 | 5 | *Psychrobacter* sp. P11G3 |
| CM003600.1 | pPspP11G3d | 5562 | 3 | *Psychrobacter* sp. P11G3 |
| CP012534.1 | pPspP11G5a | 40963 | 31 | *Psychrobacter* sp. P11G5 |
| CP012535.1 | pPspP11G5b | 14291 | 17 | *Psychrobacter* sp. P11G5 |
| CP012536.1 | pPspP11G5c | 13597 | 9 | *Psychrobacter* sp. P11G5 |
| CP012537.1 | pPspP11G5d | 9152 | 8 | *Psychrobacter* sp. P11G5 |
| CP012538.1 | pPspP11G5e | 6009 | 4 | *Psychrobacter* sp. P11G5 |
| CP012539.1 | pPspP11G5f | 5826 | 6 | *Psychrobacter* sp. P11G5 |
| CP012540.1 | pPspP11G5g | 5511 | 6 | *Psychrobacter* sp. P11G5 |
| CP012530.1 | pPspP2G3a | 23861 | 23 | *Psychrobacter* sp. P2G3 |
| CP012531.1 | pPspP2G3b | 11297 | 14 | *Psychrobacter* sp. P2G3 |
| CP012532.1 | pPspP2G3c | 9708 | 10 | *Psychrobacter* sp. P2G3 |
| CP029790.1 | pYP14a | 13712 | 17 | *Psychrobacter* sp. YP14 |
| CP029791.1 | pYP14b | 19711 | 17 | *Psychrobacter* sp. YP14 |
| CP029792.1 | pYP14c | 36270 | 35 | *Psychrobacter* sp. YP14 |
| CP029793.1 | pYP14d | 8194 | 7 | *Psychrobacter* sp. YP14 |
| CM009118.1 | unnamed1 | 15174 | 21 | *Psychrobacter* sp. Choline-02u-13 |
| CM009119.1 | unnamed2 | 7507 | 9 | *Psychrobacter* sp. Choline-02u-13 |
| CM009120.1 | unnamed1 | 15174 | 21 | *Psychrobacter* sp. Choline-02u-9 |
| CM009121.1 | unnamed2 | 7507 | 9 | *Psychrobacter* sp. Choline-02u-9 |
| CM009132.1 | unnamed1 | 14711 | 17 | *Psychrobacter* sp. MES7-P7E |
| PJBU01000018.1 | unnamed2 | 6585 | 10 | *Psychrobacter* sp. MES7-P7E |
| PJBU01000022.1 | unnamed2 | 5379 | 6 | *Psychrobacter* sp. MES7-P7E |
| CM009133.1 | unnamed3 | 7765 | 8 | *Psychrobacter* sp. MES7-P7E |
| CM009134.1 | unnamed4 | 3868 | 4 | *Psychrobacter* sp. MES7-P7E |
| CM009135.1 | unnamed5 | 2833 | 2 | *Psychrobacter* sp. MES7-P7E |
| CM009136.1 | unnamed1 | 11462 | 14 | *Psychrobacter* sp. Sarcosine-02u-2 |
| CM009137.1 | unnamed2 | 8497 | 7 | *Psychrobacter* sp. Sarcosine-02u-2 |
| CM009138.1 | unnamed3 | 7482 | 9 | *Psychrobacter* sp. Sarcosine-02u-2 |
| CM009139.1 | unnamed4 | 6971 | 7 | *Psychrobacter* sp. Sarcosine-02u-2 |
| CM009140.1 | unnamed5 | 5636 | 6 | *Psychrobacter* sp. Sarcosine-02u-2 |
| CM009141.1 | unnamed6 | 1929 | 1 | *Psychrobacter* sp. Sarcosine-02u-2 |
| NEXR01000020.1 | unnamed | 12587 | 18 | *Psychrobacter* sp. L7 |
| NEXR01000016.1 | unnamed | 4320 | 4 | *Psychrobacter* sp. L7 |
| NEXR01000017.1 | unnamed | 3650 | 2 | *Psychrobacter* sp. L7 |
| NEXR01000018.1 | unnamed | 3229 | 4 | *Psychrobacter* sp. L7 |
| JAJQQX010000003.1 | unnamed | 15383 | 18 | *Psychrobacter sanguinis* FDAARGOS_1624 |
| CP100399.1 | pPSYFG1 | 44579 | 48 | *Psychrobacter* sp. PraFG1 |
| JAJFON010000003.1 | unnamed1 | 18632 | 19 | *Psychrobacter sanguinis* FDAARGOS_1568 |
| JAJFON010000004.1 | unnamed2 | 6583 | 9 | *Psychrobacter sanguinis* FDAARGOS_1568 |
| CP085991.1 | unnamed1 | 32179 | 32 | *Psychrobacter sanguinis* FDAARGOS_1598 |
| CP085992.1 | unnamed2 | 25443 | 23 | *Psychrobacter sanguinis* FDAARGOS_1598 |
| CP085993.1 | unnamed3 | 22885 | 26 | *Psychrobacter sanguinis* FDAARGOS_1598 |
